# Comparing SVM and ANN based Machine Learning Methods for Species Identification of Food Contaminating Beetles

**DOI:** 10.1038/s41598-018-24926-7

**Published:** 2018-04-25

**Authors:** Halil Bisgin, Tanmay Bera, Hongjian Ding, Howard G. Semey, Leihong Wu, Zhichao Liu, Amy E. Barnes, Darryl A. Langley, Monica Pava-Ripoll, Himansu J. Vyas, Weida Tong, Joshua Xu

**Affiliations:** 1 Department of Computer Science, Engineering and Physics, University of Michigan-Flint, Flint, MI USA; 2 Division of Bioinformatics and Biostatistics, National Center for Toxicological Research, US Food and Drug Administration, Jefferson, AR USA; 3 Food Chemistry Laboratory-1, Arkansas Laboratory, Office of Regulatory Affairs, US Food and Drug Administration, Jefferson, AR USA; 4 Office for Food Safety, Center for Food Safety and Applied Nutrition, US Food and Drug Administration, College Park, MD USA

## Abstract

Insect pests, such as pantry beetles, are often associated with food contaminations and public health risks. Machine learning has the potential to provide a more accurate and efficient solution in detecting their presence in food products, which is currently done manually. In our previous research, we demonstrated such feasibility where Artificial Neural Network (ANN) based pattern recognition techniques could be implemented for species identification in the context of food safety. In this study, we present a Support Vector Machine (SVM) model which improved the average accuracy up to 85%. Contrary to this, the ANN method yielded ~80% accuracy after extensive parameter optimization. Both methods showed excellent genus level identification, but SVM showed slightly better accuracy  for most species. Highly accurate species level identification remains a challenge, especially in distinguishing between species from the same genus which may require improvements in both imaging and machine learning techniques. In summary, our work does illustrate a new SVM based technique and provides a good comparison with the ANN model in our context. We believe such insights will pave better way forward for the application of machine learning towards species identification and food safety.

## Introduction

Food contamination is a serious threat to public health and national well-being^1^. Pest such as insects, especially pantry and storage beetles, often find their ways into food produces (usually grains) during the storage and/or transportation^2,3^. Food products processed using insect infested raw materials and under unsanitary conditions also lead to the presence of insect contaminants in foods^4,5^. Furthermore, many species of beetle are known to be the symbiotic hosts for pathogen, with some being extremely virulent^6^. Various beetle species are also known to arrive through food products and become invasive species by out-competing the native species^7,8^. Thus, the implications of beetle contamination in food products may stretch from food safety to ecological balance. To counter such implications, food products are constantly inspected and food safety regulations are strongly enforced to keep both the consumers and the environment safe^9,10^. The most common and widely used method of food inspection involves highly trained professionals who carefully analyze food samples for insect remains (and other extraneous materials) using optical microscopy. They then match the patterns from the insect fragments with reference images to identify the insect species^2,10^. This however, is quite arduous, time consuming and needs well trained professionals. Even then, it is often challenging to correctly identify the exact species as insects from same genus often have similar appearance in pattern and minute structural features.

The last few years have seen a surge in the use of machine learning for species identification due to the technological advents in the field of pattern recognition^11–15^. Identification of species is the key in cataloging and monitoring the biodiversity which has great implications in better managing the ecology and environment^16,17^. Several reports are now available that highlight the application of machine learning in species identification, where features were first extracted to identify particular patterns or micro-structures by analyzing multiples ‘test’ images. They are then used to build a training set classifier obtained from various closely-related species^18–20^. The organisms were then identified by comparing the characteristics of these extracted features using machine learning methods that showed good accuracy for identification even to the order level^20–23^. However, most of such reports that prevail are in the context of ecology where clear and whole images of intact specimens are easily available. It is rare to find intact insect species in food products where the insect remains are often fragmented and significantly altered from the steps of food processing, which makes the species identification significantly more challenging. Hence, we explored the possibility of using advanced machine learning approaches for species identification using images of fragmented organisms that are relevant to food safety regulations.

In our previous work, we explored the feasibility of this approach to be implemented in the context of food contamination and safety regulations^24,25^. Results from the present regulatory analytical methods have shown over time that the majority of insect contaminations found in foods arise from the storage/pantry beetles^4^. Beetles (or other insects) also have hardened forewings, known as elytra, whose fragments are often found in insect contaminated food samples. Fortunately, these hard chitin-based elytra also contain particular patterns and microstructures that could be unique to the species. Hence, analyzing elytra samples to identify the beetle species using their ‘fingerprint’ of patterns seemed a logical start for us. Thus, fifteen different species of beetles that are most commonly associated with food contaminations were collected and their elytra were carefully imaged. They were then processed through MATLAB (MathWorks Inc., Natick, MA) to extract the image features. Subsequently, we trained artificial neural networks (ANN) that allowed up to 79% prediction accuracy in identifying the beetle species^25^.

Recently, SVM based methods have also been used in identifying insect species in stored grains. In one such work, Yang *et al*. used a multi-class SVM model to identify insects, based on the proportion of their wings^26^. Similar studies used size ratio of insect bodies or other anatomical features as means to identify the species^22,27,28^. However, such approaches can only be applied to ecological systems and not in food safety as beetle remains in food samples often lose their original shape, size and color due to the food processing steps. Further, these studies seldom address identifying beetle species that have similar appearance or other anatomical similarities, for belonging to the same genus^23^. Nevertheless, these works do highlight the significance of developing multiple methods for species identification, especially when the challenges are both copious and extensive.

Therefore, we explored the possibility of using support vector machine (SVM) as an alternative method for our applications. In this work, we developed an SVM based method in our quest to improve the accuracy of prediction. The multi-class SVM model was first developed to identify a beetle species from the images of its elytra fragments. This was followed by an innovative approach where binary class SVM was used to distinguish between the beetles that have similar appearances, especially for being from the same genus. Motivated by the SVM method, we also improved the ANN model that we reported earlier^25^. Both the parameters and architectures of the ANN method were thoroughly optimized in our quest to improve its accuracy. We used the same set of elytra images, feature sets and feature selection methods for both ANN and SVM based models. Our study therefore also presents a fair comparison and evaluates the performances of these two well optimized machine learning methods (ANN and SVM) in identifying the food contaminating beetle species. We believe such optimization techniques and their comparison will help future studies achieve better accuracies. We further hope that our study will also shed light on the use and influence of machine learning techniques towards the problem of species identification through pattern recognition, especially in the context of food safety.

## Materials and Methods

**Figure 1:**
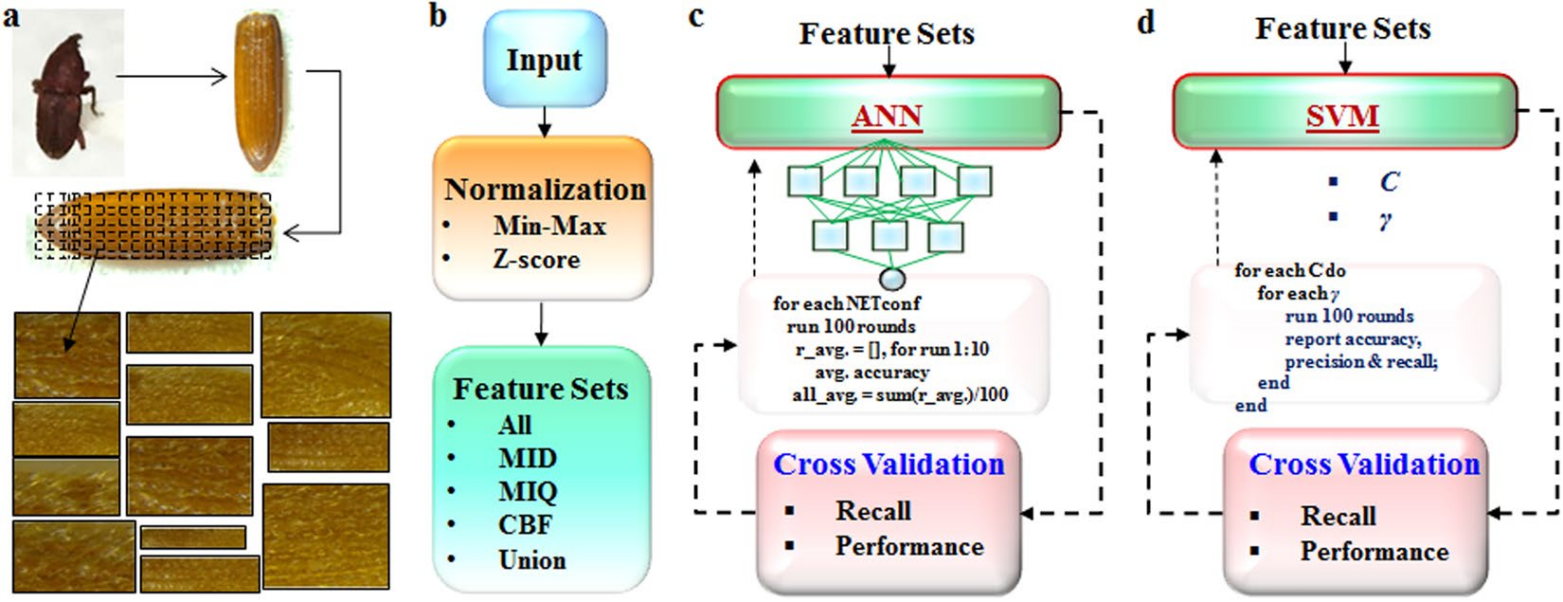
The basic experimental procedure for our work. (**a**) Steps (such as beetle collection and imaging) to obtain input images; (**b**) the input images subjected to Feature Selection Method. (**c** and **d**) and the schematic showing the training and cross validation using ANN and SVM models.

The flow of the experiments and computational steps and methods involved in our study are schematically demonstrated in Fig. 1. It started with the collection of the 15 different species of food contaminating beetles, followed by imaging their elytra. To simulate the fragmentations, each of the whole elytra images was cropped into about 100 sub-images, which were used to develop the models for species identification. The following describes the whole process in greater details.

### Imaging and data acquisition

The basic imaging and data acquisition process were described in our previous report and is schematically illustrated in Fig. 1a^25^. Briefly, we chose 15 different species of beetles that are most commonly associated with insect based food contamination. The elytra from each species were carefully isolated and several different elytra samples (5–6 per species) from each species were carefully imaged using Leica M205, at 75–100X magnification. The collected images were then enhanced through Gaussian filters and histogram-equalization. To simulate fragmentation, 100 sub-images were randomly extracted from each image of whole elytra with variable sizes and locations. This resulted in a final set of 6900 images, with an average of 460 ± 71 images for every species of beetle. Subsequently, the images were analyzed digitally for characteristic features such as the size, color, patterns, periodicity and textures, which grossly mimicked the steps and methods of taxonomical identification^3,10^.

### Image feature extraction

Identifying and recognizing the minute features or patterns within the input images is the key in identifying a species through machine learning. The process of feature extraction is thus essentially converting the microstructural patterns of the input images into their digital descriptors, a strategy that have commonly been used in species identification through pattern recognition^19–23,27–29^. To do this effectively, all the image features were categorized in 3 sets, namely Global Feature 1 (GF1), Global Feature 2 (GF2) and Local Feature (LF). The broad characteristics such as size, color, basic pattern and textures (such as lines, ridges) were categorized as GF1. More detailed features such as type of patterns (hairs, ridge lines, groves and bulges) and their periodicity were categorized as GF2. As the name describes the GF 1&2 provide a good general description of the global or general appearance of the images and provides their basic identity. Further microscopic details, such as minute changes in and around every pattern spot (such as a bulge or a grove) were categorized as a LF, as they provided information about local changes around every pattern spot and is believed to be more closely associated with the identity/order of the insects. Standard digital filtering and processing steps were used during the process of feature extraction, which have been described in detail in our previous report. Together, the GF1, GF2 and LF, allowed us to generate the classifier that essentially contained the digital ‘fingerprint’ for each beetle species. For more details on these features, please refer to our previous report^25^.

### Feature selection

Methods aiming *minimal redundancy* were used for feature selection, which allowed us to better understand the features that are more closely associated to species identification. For this, two commonly used feature selection methods were applied. The first method tries to achieve both minimal *redundancy* and *maximum relevance* (mRMR)^30^ based on mutual information of two variables, $I(x,y)$, which can be defined using joint and marginal probabilities:

1 $$I(x,y)=\sum _{i,j}p({x}_{i},{y}_{j})log\frac{p({x}_{i},{y}_{j})}{p({x}_{i})p({y}_{j})}$$

Let *S* denote the subset of features that are being sought. Then, mRMR aims to minimize redundancy, which selects the features that are mutually maximally dissimilar by the following minimization problem.

2 $${\rm{\min }}\,{W}_{I},{W}_{I}=\frac{1}{|S{|}^{2}}\sum _{i,j\in S}I(i,j)$$

On the other hand, relevance of a feature *i* to a specific class *h* needs to be maximized as follows:

3 $${\rm{\max }}\,{V}_{I},\,{V}_{I}=\frac{1}{|S|}\sum _{i\in S}I(h,i)$$

At this point, two options arise to combine the above expressions in a single maximization problem: (i) *mutual information difference* (MID) and (ii) *mutual information quotient* (MIQ) for which following expressions are optimized, respectively.

4 $${\rm{\max }}\,({V}_{I}-{W}_{I})$$

5 $${\rm{\max }}\,({V}_{I}/{W}_{I})$$

Due to the difference of two options, different rankings of the features are possible. In this study, we used the top 50 features suggested by each of these two approaches.

The second method is based on correlation and eliminates features that have low prediction ability and high degree of redundancy. This method too has two options: *correlation-based-forward* (CBF) and *correlation-based-backward* (CBB), as elimination can take place either forward or backward. It was observed from our previous study that CBF led to a better performance than that by CBB, and hence only CBF was used in this study.

### Pre-processing of features

In order to avoid over estimation, care was taken to randomly divide the sample images into a training set and a test set. About 80% of the sample images were used to build the classifier and ~20% were reserved for the cross validation. However, due to the nature of feature extraction process, about 70 samples had NaN values and hence were carefully excluded before the feature set processing. It was also observed that some samples were inclusive in one feature set and yet could be excluded in another. Thus, we had slightly different number of samples for different feature sets (MID: 6887, MIQ: 6832, CBF: 6835), but we did maintain the ratio between training sets and test sets as 80% to 20%. To address the vast difference in scales of the feature descriptors, we employed z-score normalization for each feature descriptor across all the samples: namely, we subtracted the mean from each value and divided by the standard deviation of that particular descriptor. In doing so, the original data were turned into a standard scale for each feature and evaluated in separate SVM models and ANN optimization.

### ANN Method and Parameter Optimization

In our previous report, we used 2 hidden layers with 50 nodes in ANN. In this work, we have expanded our approach to optimize the architecture. Here, we not only experimented with different number of layers, but also utilized two commonly used training algorithms for pattern recognition, *Scaled Conjugate Gradient* (***SCG***) and *Resilient Backpropagation* (***RP***). Each training algorithm, in turn, was optimized for 1, 2 and 3 hidden layers. We further augmented various numbers of nodes with an increment of 50s as represented in Table 1, and ran them with both *trainscg* and *trainrp* functions in MATLAB, giving us 36 different ANN architectures. We also performed some experiments with or without z-score to study the normalization behavior.

**Table 1:** Representation of different ANN architectures used in our work using various number of layers and nodes.

One Layer	Two Layers	Three Layers
50	(50, 50)	(50, 150, 50)
100	(50, 100)	(100, 150, 100)
150	(150, 200)	(150, 150, 150)

We initiated our experiments by using all features but subsequently utilized only the selected feature sets. Besides three independent feature selection methods, the union of 3 selected feature sets was also considered in seeking the best representation of the features. Hence, five feature sets, viz. all features, MID, MIQ, CBF, and the consensus set (CS) were subsequently used for further optimization procedure.

### SVM Method and Parameter Optimization

The SVM models are prominent for handling both linear and non-linear data. The model aims to draw decision boundaries between data points from different classes and separate them with maximum margin^30^. We used open source LIBSVM for this work due to the non-linear nature (multiple type and kind of patterns) of our feature set^31^. For the same reason, radial basis function (RBF), which is widely adopted^32^ and often outperforms other kernel functions in nonlinear classification^33^, was chosen as the kernel function to solve the classifier. The feature sets described in the ANN method (in the previous paragraph) were adopted for the SVM method as well.

As for the optimization procedure, we sought for the best γ parameter for the RBF function along with the best regularization parameter, C, for SVM. The combination of optimal γ and C values were sorted such that the influence is enough to have a decision surface without misclassifying the training set, which minimized the over-prediction.

During this stage, we aimed to develop multi-class SVM model that relies on different training and testing sets in several rounds, which is also called cross validation. This is a common approach to evaluate a predictor and can be applied in various ways such as the sub-sampling test, independent dataset test and jackknife test^34–36^. In this study, we attempted to mimic the leave-one-out test at species level with some variations. More specifically, we introduced randomness to select a particular image with its 100 sub-images for each elytra species. Then, 100 sub-images were held out for the test set in that particular round. Namely, we left out an image for a given species and tried to predict the class labels for its 100 sub-images. Since this step was repeated 100 times, 100 pairs of training and test sets were built each consisting of 5,400 and 1,500 (15 × 100) instances. It is worth noting that test sets have equal representation of each species with their 100 sub-images and we kept the same ratio between the training and the test sets. However, sub-images having NaN values in their feature vectors were removed, which resulted in slightly varying sizes for both sets.

In order to ensure the best possible optimization, we first performed a grid search across 5 orders of magnitude in logarithmic scale for varying C (from e + 01 to e + 5) and γ values (from e − 01 to e − 06). Then, for the multi-class SVM, 100 rounds of cross validation were performed. The outcomes however were not always perfectly accurate. In some cases, two species of beetles, due to their similar appearances, would be misclassified. They were defined as the ‘***difficult pairs***’ as it was difficult to distinguish between them. Another binary class SVM was then used to better distinguish between these two species of beetle. In this case, 100 rounds of cross validation were performed. For the hybrid multi-stage SVM model, first the multi-class model was run during each round of cross-validation. If the result obtained happened to be one of the *difficult pairs* (i.e. beetles species of same appearance or from same genus), then the results were fed into a binary-class SVM, which would separate the *difficult pairs*. This concatenated method was not performed and the process stopped after the multi-class SVM if the outcome species is *not* from a difficult pair.

### Metrics Used to Evaluate the Prediction Quality

In order to compare aforementioned classification efforts, we calculated a confusion matrix for each round of cross validation from which we derived the counts for true positive (TP), false positive (FP), true negative (TN), and false negative (FN) cases. Due to the nature of multi-classification, we computed these values for each class label (species) from an *M* × *M* confusion matrix as follows.

Let *C*_*M*×*M*_ be the confusion matrix for *M* species where rows represent the actual class label and columns stand for the predicted labels. Then, for a given class label *i*,

6 $$FP=\sum _{j=1}^{M}{C}_{ji}-{C}_{ii}\,$$

7 $$TP={C}_{ii}$$

8 $$FN=\sum _{j=1}^{M}{C}_{ij}-{C}_{ii}\,$$

9 $$TN=\sum _{i=1}^{M}\sum _{j=1}^{M}{C}_{ij}-(FP+TP+FN)$$

Once we obtained the values above, we evaluated the performance of each approach by the following five metrics: (i) *Accuracy*, (ii) *Sensitivity*, (iii) *Precision*, (iv) *Specificity*, and (v) *Matthew’s correlation coefficient (MCC)*, which are commonly used in biological research^37–41^ and defined as below.

10 $$Overall\,Accuracy=\frac{{\sum }_{i=1}^{M}T{P}_{i}}{{\sum }_{i=1}^{M}T{P}_{i}+F{N}_{i}}\times 100 \% $$

11 $$Sensitivity=\frac{TP}{TP+FN}\times 100 \% $$

12 $$Precision=\frac{TP}{TP+FP}\times 100 \% $$

13 $$Specificity=\frac{TN}{TN+FP}\times 100 \% $$

14 $$MCC=\frac{(TP\times TN)-(FP\times FN)}{\sqrt{(TP+FN)\times (TP+FP)\times (TN+FN)\times (TN+FP)}}$$

Since our work primarily relies on cross validation, we first computed the overall accuracy of each model by Eq. 10 for every round and reported the global statistics such as mean and standard deviation in table format. As for the performance evaluation per species (across all rounds of cross validation), we used the remaining four metrics listed through Eqs 11 to 14. However, due to the virtue of our study design, Accuracy per species essentially becomes the same as *Sensitivity* as they both refer to the measure of true predictions for a given class.

## Results

**Figure 2:**
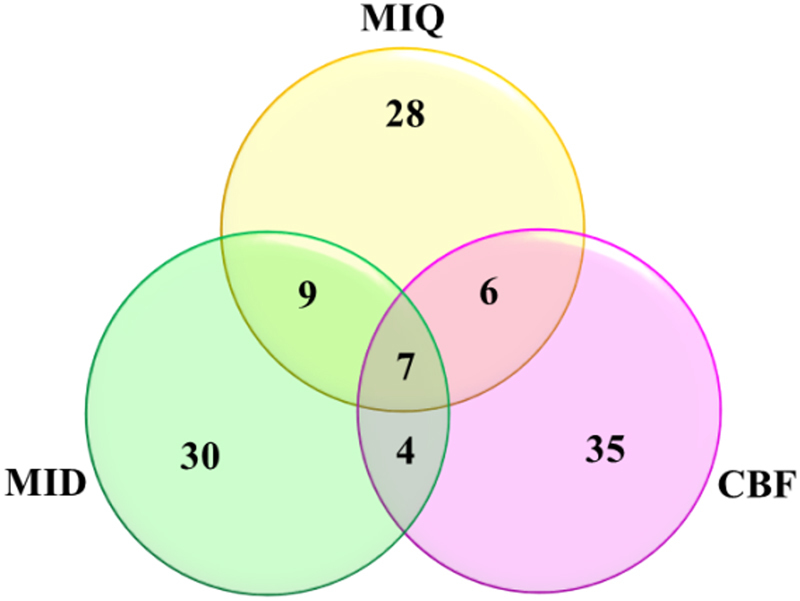
Overlapping of features for various feature selection methods. Only 7 features were found to be selected by all three methods.

A total of 625 features were generated from the elytra images (Supplementary Table S1). Feature selection methods were applied to extract the top ~50 features in each method, (MID & MIQ 50; CBF 52). The union of three feature sets yielded 119 features in the CS, as shown in Fig. 2 (Supplementary Table S2). It is worth noting that the feature sets have only 7 features in common and the majority of them are unique to that particular set. This could be due to the fact that the nature of the patterns/features on the beetle elytra can be quite diverse based on the genus and/or the species. Also, some of the features were partially correlated and redundant as they were extracted and we never intended to make them uncorrelated; hence statistical enrichment methods may not necessarily highlight the common features that bear relevance to their identities.

For the SVM method, each feature set, the best values for *C* & *γ* were sought by developing 100 rounds of SVM models and testing them on the corresponding test sets. Table 2 summarizes the identification accuracies that were averaged across 100 rounds for each feature set for the best (*C*, *γ*) pair. The MID, MIQ and CBF showed a performance of 81, 83 and 79% respectively with their best parameters (Table 2), with the consensus set (CS) yielded 85% accuracy. All feature selection methods including the consensus set did not show significant improvement than using all 625 features (84%). This reflects that the feature selection method may have limitations in improving the performance. However, careful selection of some features, such as through the CS model, that has significantly less number of features, will provide a more efficient model, when dealing with a large data set.

**Table 2:** The significance of feature selection on the performance for the multi-class SVM model.

Feature Set	*C*	*γ*	Overall Accuracy	Std. Dev
All (625)	1e + 02	1e − 04	0.84	0.048
MID (50)	1e + 03	1e − 05	0.81	0.050
MIQ (50)	1e + 01	1e − 03	0.83	0.046
CBF (52)	1e + 02	1e − 03	0.79	0.044
CS (119)	1e + 01	1e − 03	0.85	0.044

**Figure 3:**
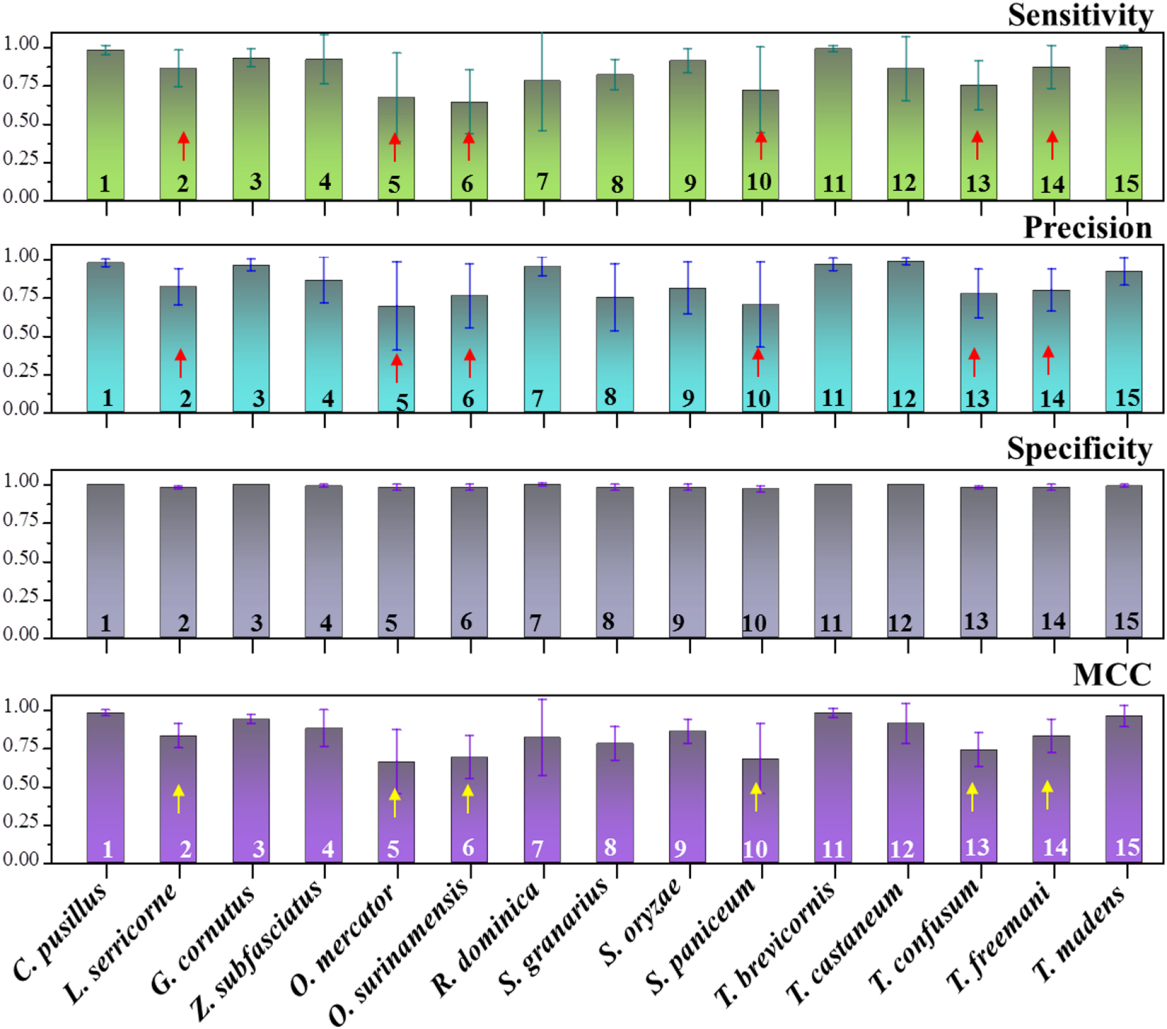
Prediction performance as *Sensitivity*, *Precision*, *Specificity* and *MCC* values for the identification of all 15 species of beetles. It can be noted that some species of beetles could be identified with better confidence level than others. However, beetles such as species 2&10; 5&6 (of genus *Oryzaephilus*) and 13&14 (of genus *Tribolium*) were difficult to be identified (indicated by the arrows).

The CS model was thus carried forward for the species identification using the multi-class SVM method. The prediction performance was assessed by 4 metrics namely *Sensitivity*, *Precision*, *Specificity* and *MCC*, which have collectively been presented in Fig. 3 for all the 15 different beetle species. Almost near perfect *Sensitivity* values were obtained for species 1, 3, 4, 9, 11 & 15. Similarly, excellent *Precision* (positive prediction) and *MCC* values could be observed for the species 1, 3, 4, 7, 11, 12 & 15. On the contrary, the *Precision* values were quite low for species 2, 5, 6, 10, 13 and 14, with species 5&6 yielding less than 80%. The *Sensitivity* and *MCC* values were also not high for species 2, 5, 6, 10, 13 & 14. Overall, the results from the multi-class SVM method suggest that some beetle species are more difficult to identify (and distinguish) than the others as indicated by arrows in Fig. 3. For example, our model falls short in distinguishing between species 2 & 10; 5 & 6 or 12, 13 & 14. This seems logical, since the elytra patterns of species 2 & 10 (*G. cornutus* & *S. paniceum*) have very similar appearance. This is even more so for species 5 & 6 (*O. mercator* & *O. surinamensis*) and species 12, 13 & 14 (*T. castaneum*, *T. confusum* & *T. freemani*) which being from the same genus, are known for their exceedingly similar appearances that sometimes even confuse trained entomologists^3,4^.

The under-performance of the multi-class model to distinctively identify beetles of similar elytra patterns or of same genus presented us with the challenge of separating between the ‘*difficult pairs*’. Hence, binary-class SVM model was implemented to these three sets of *difficult pairs*. Table 3, presents the best values of *C* and *γ* along with the average accuracies and standard deviations for 100 rounds of cross validation, for the binary class SVM model.

**Table 3:** Accuracy, C and γ values for the binary class SVM model to differentiate between the difficult pair.

Species Pair	*C*	*γ*	Accuracy	Std. Dev
S2–S10	1e + 02	1e − 05	0.87	0.08
S5–S6	1e + 02	1e − 05	0.79	0.13
S13–S14	1e + 02	1e − 03	0.87	0.11

It can be noted that the binary-SVM did improve the accuracy of prediction and this approach could distinguish between the *difficult pair* species 2&10 as it has improved the accuracy to 87%. The same improvement was also observed for species 13&14, as the accuracy increased to 87%. This is significant, as our model can now distinguish between two species from the same genus, (i.e. *T. confusum* & *T. freemani* of genus *Tribolium*), which is regarded difficult even for entomologists when looking only at their elytra patterns. The improvement however falls short for the ‘*difficult pair*’ species 5&6 (*O. mercator* & *O. surinamensis* of genus *Oryzaephilus*). This could be due to extensive similarity of patterns between two species can be easily confuse one from the another^42^.

**Figure 4:**
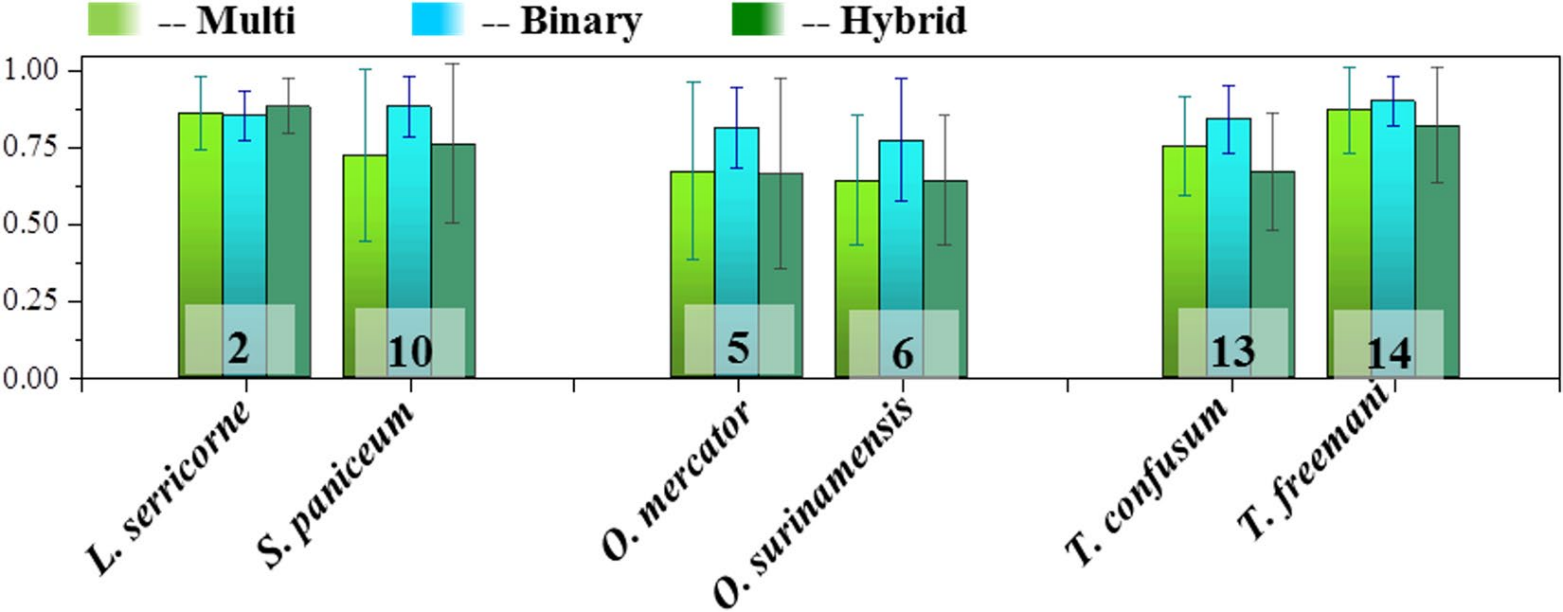
Performance comparison for the original Multi-class and Hybrid SVM models in terms of their Accuracy values for the difficult pairs. The Hybrid Model that was aimed to combine the advantages of Multi-class and Binary models failed to outperform the original Multi-class model.

The improvement of predictions in the species identification (except for one pair) encouraged us to consider a hybrid model, where the feature set is first solved with a multi-class SVM model, followed by a binary-class model if the former predicts a ‘*difficult pair*’. We hypothesized that this multi-stage, hybrid model would probably be able to combine the best of both models, i.e. predict the correct identity from any number of species (the advantages of the multi-class SVM) and with a reasonably good accuracy even for the *difficult pairs* (the key feature of the binary class SVM). Figure 4 compares the accuracy values for multi-class and hybrid multi-stage (1^st^ multi-class then binary) SVM models. It was indeed possible to get slightly better accuracy (88%) when the outcome of the 1st step yields the difficult pair 2/10. This, however, was not the case when the 1st step of the hybrid model yielded either 5/6 or 13/14. One possible reason is that the features used for the multi-class model may not be as relevant for the binary class models. For example, the features arising from the structural patterns (that are of Global Feature 2) are almost identical for 13&14 (*T. confusum* & *T. freemani* being from the same genus *Tribolium*) and can only be distinguished by the feature set arising from their color (Global Feature 1). Thus, it may be possible that the subtle differences in their colors are either not translated onto the classifier or may have been lost during the feature selection process^28,43^.

Compared to our previously reported ANN method, the accuracies of our newly developed SVM were found to be slightly better. This improvement did come from our strategy of screening and optimizing a wider range of the parameters and using the CS. This encouraged us to better optimize our previously developed ANN method in order to improve its accuracy. Thus, efforts were made in optimizing both its training algorithm and network architecture. Since the CS feature sets yielded the best results and even outcompeted the all feature set in SVM, hence we used only this feature set to optimize the ANN method.

First, we optimized the model by employing different training algorithms with varying number of hidden layers and hidden nodes. Training algorithms, ***SCG*** and ***RP*** were applied with ***trainscg*** and ***trainrp*** functions in MATLAB. For each algorithm, the network architecture was varied for 1, 2 and 3 hidden layers, with the nodes varied from 50 to 150 at increments of 50 in each hidden layer. As shown in Table 4, the *trainscg* function performed better than the *trainrp* function in all architectures. The best accuracies that we could obtain were slightly above 79% for all three layers. Additionally, we observed that slight improvements could be made by increasing the number of nodes. However, increasing the number of nodes even up to 400 only marginally increased the accuracy to 80% for 2 and 3 layer architectures.

**Table 4:** Accuracy values for ANN models with different parameters.

No. of layers	No. of Nodes	Function	Overall Accuracy
1	50	*trainscg*	0.77
	50	*trainrp*	0.70
	100	*trainscg*	0.79
	100	*trainrp*	0.69
	150	*trainscg*	0.79
	150	*trainrp*	0.67
2	(50, 50)	*trainscg*	0.78
	(50, 50)	*trainrp*	0.71
	(50, 100)	*trainscg*	0.78
	(50, 100)	*trainrp*	0.70
	(100, 150)	*trainscg*	0.79
	(100, 150)	*trainrp*	0.51
3	(50, 150, 50)	*trainscg*	0.78
	(50, 150, 50)	*trainrp*	0.58
	(100, 150, 100)	*trainscg*	0.79
	(100, 150, 100)	*trainrp*	0.52
	(150, 150, 150)	*trainscg*	0.79
	(150, 150, 150)	trainrp	0.43

We also investigated the normalization (with and without z-score) conditions which did not show much change in accuracies as highlighted in Table 5. With all the variations in architectures and optimization of parameters the overall accuracy only increased marginally from our previous report of 79%, with the maximum of 80% which was obtained for 2 layer architecture with (350, 400) nodes. Thus, we concluded that accuracy levels can hardly be stretched beyond 80%, using this method with the given set of features or input images.

**Table 5:** The effect of z-score normalization.

No. of nodes	without z-score	z-score
(50, 50)	0.78	0.78
(50, 100)	0.78	0.78
(100, 150)	0.79	0.79

Similar to our previous report and the SVM method, the individual performance for the ANN method also varied according to the species (Supplementary Figure S1). While some species such as species 1, 3, 4, 11 & 15 yielded good prediction performances (for all 4 metrics namely: *Sensitivity*, *Precision*, *Specificity* and *MCC*), some others such as 2, 5, 6, 8, 10, 13 & 14 fell short from the overall average. The reason for this can again be attributed to the entomological similarities between the species. The beetle species 2&10; 5&6; 8&9 and 13&14 are *difficult pairs* and the method did not perform well in these cases and failed to appreciably distinguish between the species from the same genus. However, our collective results do highlight that both the ANN and SVM models are efficient enough to predict the species identity at the genus level. This is significant since accurate identification of the genus of the beetle may be sufficient in several applications. For example, in many regulatory practices of food safety, the goal is often to screen a larger sample volume than have detailed characterizations of a very small sample set. In such applications, where fast, efficient and inter-mediate screening is necessary, perhaps an initial genus level accuracy is a welcome relief, which the ANN and SVM models are quite capable of providing.

## Discussion

The broader objective of this work is to develop a machine learning method that would eventually identify food contaminating beetles even to the species level. We plan to achieve this by training machines to recognize a set of patterns or features that are associated with a particular beetle species. The classifier of these features sets, obtained from the images of beetle elytra, could be solved using machine learning algorithms such as ANN and SVM, each having its own advantages and disadvantages^14,15,21,22^. In our previous work, we explored the feasibility of machine learning in the context of food safety using an ANN based method to identify the beetle species from the images of their elytra fragments. This exploratory work provided us with the insight of the extent and nature of the complexity associated with this problem. We realized that it is only prudent to explore different methods to counter such a convoluted problem. Therefore, we investigated upon a SVM based method not just to identify the beetle species from their elytra fragments but also to improve on the accuracy of prediction.

Due to the processed non-linear nature of the feature set, the RBF kernel function was used in our SVM model, as such approaches has been reported in similar situations^21,22,27,44^. The feature set was subjected to various feature selection methods to narrow down more prominent features before using a SVM model. This enrichment step, especially using the CS approach, did help us achieve excellent genus level identification. But the use of the CS, which is the union of MID, MIQ and CBF methods, did not drastically increase the accuracy (only 2% more than MIQ). Our results thus suggest that the key in improving the accuracy may lie in obtaining better quality images and extracting as many features as possible from them. The classifier with a large feature set can then be solved using RBF kernel function as it is suitable for higher dimensions and certainly has the potential to manage several different feature possibilities along with multiple types of images^32,45,46^. The present work also highlight that such a strategy could address the challenge in distinguishing between the difficult pairs, which could not be achieved previously^21,22,25,28,47^. The accuracy was found to be ~87% for the species from the same genus, which was also higher than the ANN methods that we previously developed^25^.

We believe it can be improved further when we use a larger data set with more features which is currently underway. But before proceeding to such extensive and elaborate experimental efforts, it was important to demonstrate that the SVM method could provide an accurate and robust solution for this problem, which primarily is the essence of this work as it helps lay out a solid foundation to our future efforts.

One can argue that this increase in accuracy could be implicated to the feature selection method along with the detailed optimizations in the SVM method. To answer such a question, we embarked on improving the ANN method using the same strategies, namely the use of feature selection method and the detailed optimization of its parameters. We believe that the features selected through the CS model is the most rational means to minimize the feature numbers, as it outperforms three separated feature sets in ANN as well. The nature of the training function also influenced the accuracy values, as the *trainscg* function was found to perform better than the *trainrp* function for all the architectures. In spite of all the optimization processes, the accuracy did not improve significantly from the one obtained in our previous study, the best incrementing only to 80% from ~79%. The individual performance for each species was found to vary amongst the species with some performing poorly due to their similarities in appearances with the other. Those species that appear similar to each other (*difficult pairs*) could not be separated using the ANN based method. Moreover, for the ANN method, the accuracies were quite close for the data set treated with or without z-score normalization, which suggests that the normalization was not necessary for the ANN method.

**Figure 5:**
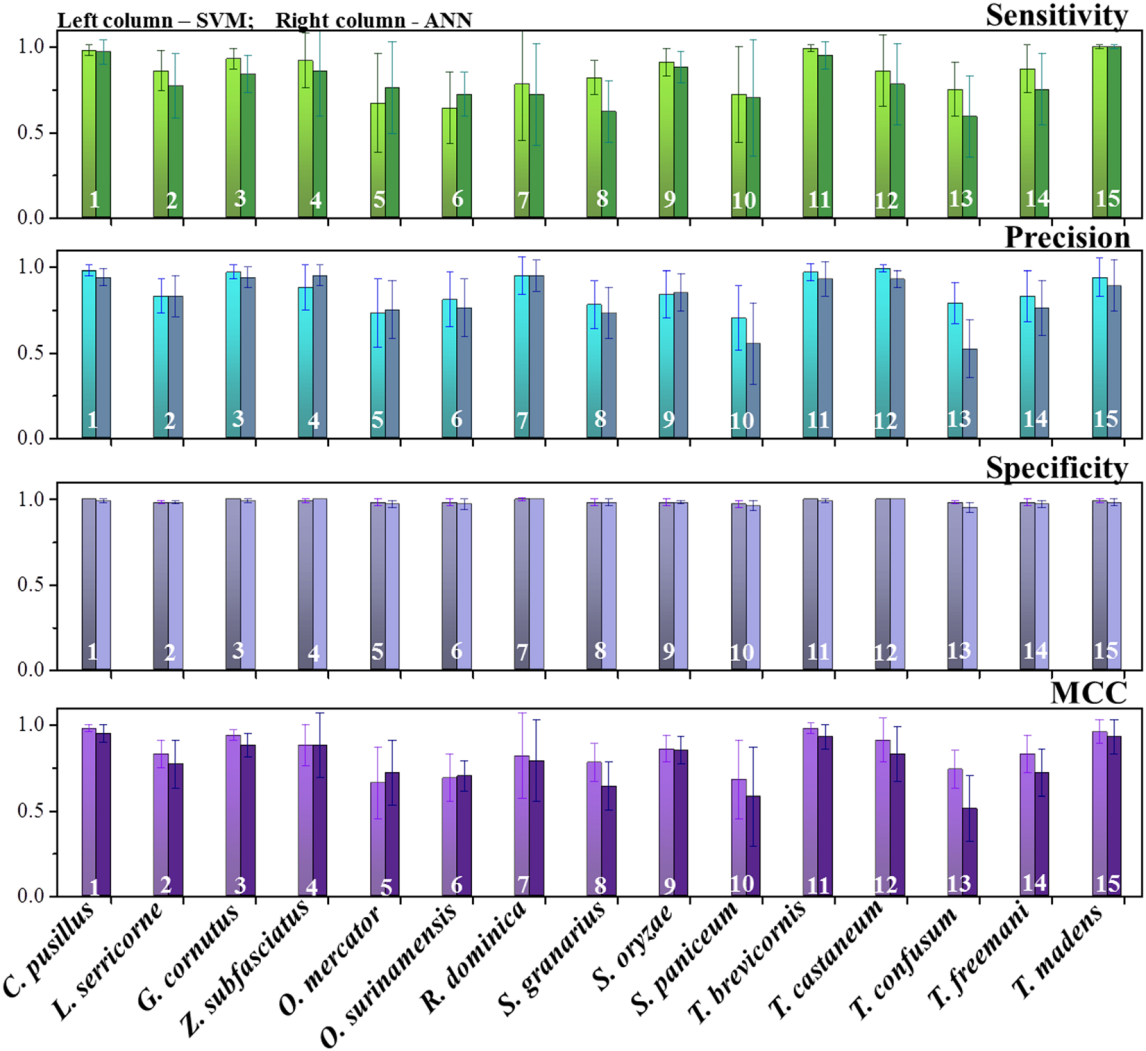
Comparison the performance metrics between ANN and SVM methods for individual beetle species. It can be noted that both SVM and ANN methods are quite comparable to each other, with each having its own advantages.

The proper optimization and similarity of treatments of both the SVM and ANN method therefore also allowed us to compare these computational methods in our context. We found that the SVM slightly outperforms ANN for the species recognition for our application using the present data set. The exact reason for this improvement is difficult to pin-point and could simply be due to better parameter selection or the diverse and non-linear nature of the data set or both. It could also be due to the fact that the SVM converges on a global minimum and allows a better tolerance to the noise (deviation from the pattern that often inherently associated with the original images) therefore might be slightly more robust for a large set of features^30,44,46^. Thus, from the perspective of convergence and robustness, SVM may have certain advantages over ANN. Figure 5 compares the accuracy values using ANN and SVM methods for each species of beetle. It can be noted that the SVM method marginally out-performs the ANN for most beetles, except for species 5&6, i.e. the beetles of genus *Oryzaephilus*, as indicated by the red arrows. This could be due to the marked similarities in their elytra patterns. Other than the minute difference in their coloring, their elytra are almost identical to each other^4^. Such differences in features may have received slightly more weightage in the ANN method compared to the SVM^5,19,23^. It could also simply be an anomaly that may clear out when larger numbers of better quality images are used for both the methods. But such anomalies do highlight the importance of developing multiple methods for a multi-facetted problem like this. They each have their own advantages and disadvantages for a particular set of images and their mutual comparison could show us the best way forward, which essentially is the rationale behind the present study.

While we achieved high overall accuracies through SVM and ANN models, the *difficult pairs* have remained as a challenge, which might be due to the high entomological similarities within those pairs. The limited number of specimen images per species not only restricted us in sample sizes, but also made it harder to distinguish those difficult pairs. Besides the quantity of images, quality has appeared to be another issue that might have introduced artifacts in the feature extraction stage. Even though we compared prediction models under the same conditions, having actual beetle fragments rather than sub-images could be closer to a real-world scenario. As indicated in our earlier work, some other food storage beetles are lacking in our current repository. Hence, collection, storage, and analysis of high quality microscopic images for a larger variety of beetle fragments remain as some of the future works.

## Conclusion

In summary, both ANN and SVM could be used to identify the species of food contaminating beetles from the patterns on their elytra fragments. The multi-class SVM method was found to be a good strategy for the beetle species identification. It had average overall accuracy of 85%, when features selection methods were consolidated with the consensus approach. For individual species, it showed excellent genus level accuracy but could not distinguish between the beetles with very similar appearance (*difficult pair*). To address this, an additional binary-SVM method was developed that could improve the accuracy up to 87% for some *difficult pairs*. However, their concatenated hybrid model did not perform as well as either (multi-class or binary class) of SVM models. Similar to the SVM model, our previously developed ANN model was also subjected to feature selection methods followed by extensive optimization of it parameters. But the optimization of both the architecture and the parameters hardly improved the accuracy, with the overall average reaching only to ~80%. At an individual level, the SVM method worked better for most of the beetle species expect of the species 5&6 of genus *Oryzaephilus*. This anomaly could be due to the vivid similarity between their elytra patterns and only highlights that one method may not be sufficient to completely address this problem. Hence, we believe the comparative study between these two machine learning methods builds an excellent platform for the future studies in this area, which are currently underway.

### Disclaimer

The views expressed in this work are those of the authors only and do not necessarily express the views/policies of the FDA.

## Electronic supplementary material

Supplementary Information

Supplementary Table S1
