# Supplementary material for: Comparing SVM and ANN based Machine Learning Methods for Species Identification of Food Contaminating Beetles
Source: Sci Rep. 2018 Apr 25;8:6532. doi: 10.1038/s41598-018-24926-7 (PMC5917025; doi:10.1038/s41598-018-24926-7)
Supplement: Supplementary file 1 — Supplementary Information [file 41598_2018_24926_MOESM1_ESM.pdf]

Comparing SVM and ANN based Machine Learning Methods for Species Identification of Food Contaminating Beetles

Halil Bisgin, Tanmay Bera, Hongjian Ding, Howard G. Semey, Leihong Wu, Zhichao Liu, Amy E. Barnes, Darryl A. Langley, Monica Pava-Ripoll, Himansu J. Vyas, Weida Tong, Joshua Xu

Disclaimer:

The views expressed in this work are those of the authors only and do not necessarily express the views/policies of the FDA.

Supplementary Information:

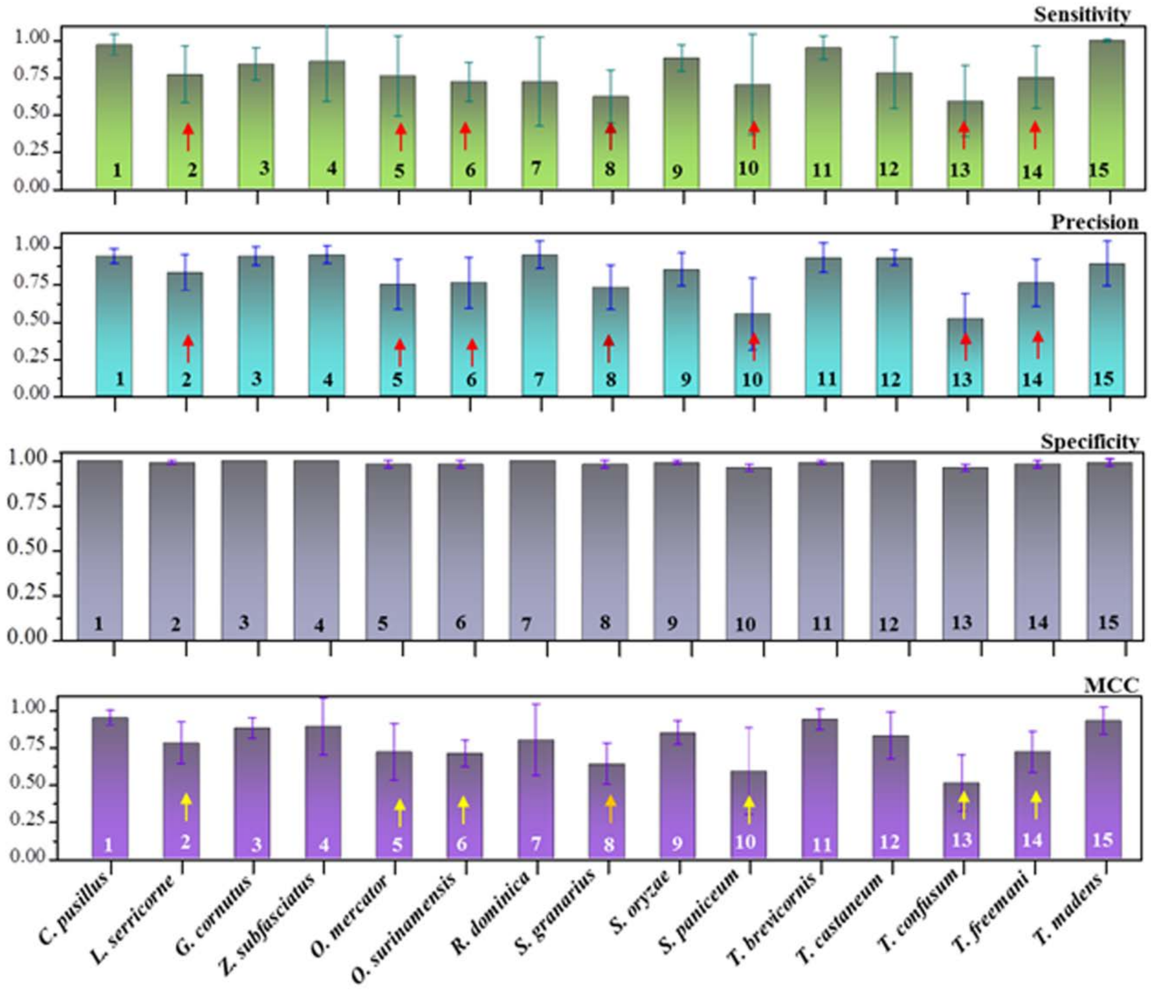

Supplementary Figure 1: Classification performance as *Sensitivity, Precision, Specificity and MCC* values for the identification of all 15 species of beetles for ANN method. It can be noted that other than species 2&10; 5&6 (of genus *Oryzaephilus*) and 13&14 (of genus *Tribolium*), species 8 (*S. granarium*) was also performed less accurately in this method.

Table S1: The feature matrix for 6900 sub-images with numerical species indices in the last column, attached as a separate file in zip compressed format.

Table S2: The list of numerical feature indices for 4 feature subsets used to build ANN and SVM models. Features #1-#142 are from Global Feature Set 1 (GF1) and color coded in light blue. Features #143-#210 are from Global Feature Set 2 (GF2) and color coded in light green. Features #211-#625 are from Local Feature Set (LF) and color coded in light brown.

**CBF**	**MID 50**	**MIQ 50**	**CS**
1	20	14	1
3	21	24	3
5	22	115	5
163	24	116	14
164	25	117	20
174	116	126	21
177	117	134	22
187	120	142	24
196	125	150	25
205	129	158	115
206	133	163	116
207	134	165	117
208	135	167	120
210	136	182	125
223	163	192	126
250	167	208	129
278	174	209	133
294	197	215	134
305	199	235	135
352	200	239	136
365	207	253	142
372	316	278	150
374	332	288	158
375	333	301	163
378	337	319	164
379	338	333	165
382	389	384	167
383	408	393	174
404	411	404	177
407	415	406	182
408	416	408	187

410	417	410	192
411	418	411	196
414	427	414	197
417	437	417	199
431	438	423	200
437	442	437	205
438	443	443	206
439	481	447	207
442	482	448	208
443	492	457	209
446	493	460	210
447	497	493	215
449	498	514	223
450	514	542	235
494	612	555	239
521	613	559	250
538	614	581	253
550	621	613	278
551	622	622	288
591			294
613			301
			305
			316
			319
			332
			333
			337
			338
			352
			365
			372
			374
			375
			378
			379
			382
			383
			384
			389

			393
			404
			406
			407
			408
			410
			411
			414
			415
			416
			417
			418
			423
			427
			431
			437
			438
			439
			442
			443
			446
			447
			448
			449
			450
			457
			460
			481
			482
			492
			493
			494
			497
			498
			514
			521
			538
			542
			550

			551
			555
			559
			581
			591
			612
			613
			614
			621
			622
